# Genome composition and phylogeny of microbes predict their co-occurrence in the environment

**DOI:** 10.1371/journal.pcbi.1005366

**Published:** 2017-02-02

**Authors:** Olga K. Kamneva

**Affiliations:** Department of Biology, Stanford University, Stanford, California, United States of America; Memorial Sloan-Kettering Cancer Center, UNITED STATES

## Abstract

The genomic information of microbes is a major determinant of their phenotypic properties, yet it is largely unknown to what extent ecological associations between different species can be explained by their genome composition. To bridge this gap, this study introduces two new genome-wide pairwise measures of microbe-microbe interaction. The first (genome content similarity index) quantifies similarity in genome composition between two microbes, while the second (microbe-microbe functional association index) summarizes the topology of a protein functional association network built for a given pair of microbes and quantifies the fraction of network edges crossing organismal boundaries. These new indices are then used to predict co-occurrence between reference genomes from two 16S-based ecological datasets, accounting for phylogenetic relatedness of the taxa. Phylogenetic relatedness was found to be a strong predictor of ecological associations between microbes which explains about 10% of variance in co-occurrence data, but genome composition was found to be a strong predictor as well, it explains up to 4% the variance in co-occurrence when all genomic-based indices are used in combination, even after accounting for evolutionary relationships between the species. On their own, the metrics proposed here explain a larger proportion of variance than previously reported more complex methods that rely on metabolic network comparisons. In summary, results of this study indicate that microbial genomes do indeed contain detectable signal of organismal ecology, and the methods described in the paper can be used to improve mechanistic understanding of microbe-microbe interactions.

## Author summary

It is still unknown to what extent ecological associations between microbes, as measured by co-occurrence of different taxa in 16S rRNA surveys, can be explained, or predicted, using composition and structure of microbial genomes alone. Here I introduce two new genome-wide, pairwise indices for quantifying the propensity of microbial species to interact with each other. The first measure quantifies similarity in genome composition between two microbes. The second measure summarizes the topology of a protein functional association network built for a given pair of microbes and quantifies the fraction of network edges crossing organismal boundaries. I then study the ability of two newly proposed and two previously reported indices to explain variation in microbial co-occurrence. All four measures are significantly correlated with co-occurrence of microbes even when accounting for evolutionary relationships between the species. One of the newly developed indices outperforms previously proposed ones and explains up to 3.5% of the variance in co-occurrence. In summary, the indices described here are able to detect ecological associations between species using only their genomic information; however, additional methods are needed to provide more reliable genomic tools for microbial ecology.

## Introduction

Due to the rise of polymicrobial infections [1], the potential of community replacement therapy in preventing infections after antibiotic treatment [2–4], and the developing interest in microbiome engineering [5,6], there is a pressing need to better understand the mechanisms behind microbial community assembly and function. Unfortunately, the processes that govern complex communities of microorganisms remain poorly understood. Below, I describe the two canonical approaches used in microbial ecology to predict interactions between microbes and explain their limitations.

### Phylogenetic marker-based approaches in microbial ecology

Classical approaches for characterizing microbe-microbe interactions include environmental surveys where the presence or abundance of different species in the community is estimated from the presence or abundances of lineage specific 16S rRNA or other phylogenetic markers [7,8]. These types of data collected from a variety of different but related habitats [9–11] or from the same habitat across time or space [12,13] are used to understand microbe-microbe interactions. The interactions are inferred from concerted changes in organismal abundance or patterns of species co-occurrence. While 16S rRNA based approaches to the problem are informative, they do not provide a clear way to understand the molecular mechanisms of inferred dependencies between the species.

### Genomics-based approaches in microbial ecology

While 16S rRNA based approaches do not lead mechanistic understanding of inferred patterns of microbe-microbe interactions, it is known that such interactions are driven by microbial metabolism and physiology: bacteria compete for essential nutrients [14,15], form food chains [16], and influence each other via secondary metabolites [17] and signaling molecules [18]. However, the extent to which global genome composition and structure influences organismal ecology remains undetermined, and only recently have researchers attempted to use genomics-based approaches to characterize microbial communities and their governing molecular principles.

The most popular currently existing genomics-based approaches for predicting relationships between microbes were developed within “reverse ecology” framework [19,20]. This framework produces indices measuring metabolic complementarity (the fraction of biochemical compounds predicted to be necessary for the metabolism of one microbe but synthesized by another) and metabolic competition (the fraction of biochemical compounds predicted to be necessary for the metabolism of both microbes), which can be used to evaluate how two given microorganisms might interact metabolically [21]. While these metrics are well regarded and have been used to study microbe-microbe interactions in human gut and other human associated habitats [21–23], it is not known to what extent they are able to explain ecological associations between microbial species.

Metabolic competition and complementarity indices are constructed upon the Kyoto Encyclopedia of Genes and Genomes (KEGG) biochemical pathway annotations, which are available only for some proteins (often a small fraction) in any given genome. Additionally, level of KEGG pathway annotation depends heavily on how extensively a given microorganism has been studied. These are two potential limitations to using KEGG microbial comparative genomics in general and for understanding microbial ecology with metabolic indices in particular.

An alternative to using KEGG pathways is to assess protein functional associations with genome content-based methods. These methods infer functional associations between proteins using measures derived from a number of distantly related genomes, summarizing information on their composition and structure [24]. Well-known genome content-based methods for predicting protein functional associations include phyletic profile, gene neighbor, and gene fusion [24]. The gene neighbor approach is built upon the observation that functionally related proteins tend to be encoded next to each other in various microbial genomes. Co-encoding is driven by the contribution of horizontal gene transfer to microbial genome evolution [25] as well as some aspects of transcriptional regulation in prokaryotes [26]. The gene neighbor approach has been shown to outperform other methods when evaluated using EcoCyc complexes and pathways data [27]. While composite methods incorporating information from several genome content-based prediction strategies have been proposed, they were not found to provide a significant advantage over the gene neighbor method alone [28].

While the gene neighbor method or other genome content-based methods do not pinpoint the exact molecular mechanisms of functional associations between the proteins, they have been successfully used to predict novel cellular systems [28,29], biosynthetic gene clusters producing secondary metabolites [30,31], CRISPR associated genes [32], and novel genetic components of known metabolic pathways [33,34]. The success of genome content-based methods in understanding the biology of individual genomes suggests that these methods could be useful in evaluating functional relationships between the proteins within supra-genomes of microbial communities as well [35]. Additionally, genome content-based inference about functional associations between proteins should be less affected than KEGG pathways by how well an organism is understood, which mitigates at least one limitation of using KEGG annotation.

### Aims of the study

In this study I aim to address two following points: (1) to develop new genomic indices for quantifying propensity of the microbes to interact with each other using gene neighbor method for predicting functional associations between proteins; (2) to understand to what extent microbe-microbe interactions, represented by microbial co-occurrence, can be explained using genomic information alone. I also evaluated how well newly developed genomics-based methods can predict microbial co-occurrence in comparison to already existing ones.

## Results

### Gene neighbor-based predictions incorporate a large fraction of genes across bacterial genomes than KEGG pathways

To better understand what fraction of ORFs (Open Reading Frames) across variety of microbial genomes is annotated with KEGG pathways information or using gene neighbor-based predictions I surveyed. 308 microbial genomes from ecological dataset 2 (described later). Results indicate that between 10% and 65% of the ORFs are included in the KEGG pathways data from IMG JGI (S1A Fig). On the other hand, using gene neighbor-based predictions allows for the incorporation of information from a much larger fraction of genes encoded in each genome. Putative pathways predicted using clustering of protein functional association networks (also called “clusters of functionally linked genes”, or “gene sets” throughout the manuscript) incorporate between 35% and 95% of all ORFs (S1B Fig). Proportion varies across organisms and depending on minimal allowed gene set size.

### New metrics for quantifying associations between microbes

#### Genome content similarity index

**Fig 1:**
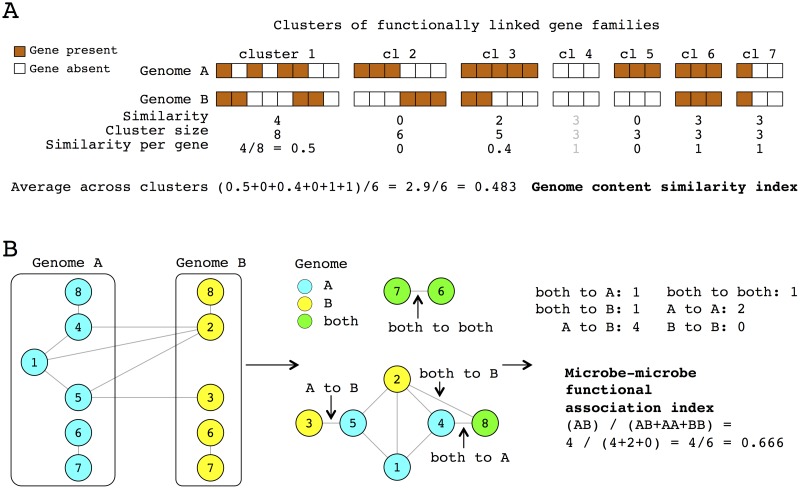
An illustration of how new genomics-based indices are computed. (A) Genome content similarity index. In case of gene set 1 there are four gene families which are absent or present in both genome A and genome B, resulting in similarity value of 4 for this gene set. In total gene set 1 contains 8 gene families, which means on average 0.5 of them have the same presence/absence state. This way gene set specific similarity per gene was calculated for each gene set, in current illustration there are 7 of them. Further, to produce genome-wide summary scores are averaged across gene set of appropriate size and represented in at least one of the genome (see text for details). (B) Microbe-microbe functional association index. Genomes of two species (A and B) encode genes from 6 and 5 gene families respectively, three gene families are encoded exclusively in genome A (1, 4 and 5), two exclusively in genome B (2 and 3), and three in both genomes (6, 7 and 8). These three categories label the nodes of the protein functional association network. Edges connecting gene families are classified in 6 classes as shown on the figure. Edges connecting gene family encoded in only genome A to gene family encoded in only genome B would have to cross organismal boundary in order to exist within the network of two-species (A and B) community.

I designed the “genome content similarity index” to capture the overall similarity of genome content of any two given microbes. The central idea of using this metric is that microbes with more similar genomes might ether exclude each other through competition or co-occur with each other due to habitat filtering. This index was calculated for every relevant pair of genomes as illustrated in Fig 1A and explained in details in Materials and Methods section.

#### Microbe-microbe functional association index

I design the “microbe-microbe functional association index” to capture potential for interaction between proteins encoded in genomes of two microbes if there was no boundary between them. This metric was calculated for every relevant pair of genomes as illustrated in Fig 1B and explained in details in Materials and Methods section.

The approach of ignoring organismal boundaries and treating a community of microbes as one organism is consistent with the idea of a metagenome of a supra-organism, which is commonly used in microbial ecology [36]. The idea of a supra-organism is mechanistically justifiable through the presence of diffusible molecules which connect metabolic networks of single individuals [37], release of all of the metabolites and macromolecules in case of cell lysis [38], and the existence of extracellular proteins carrying out their functions on the outside of the cell [39], in addition to other possibilities.

### Empirical distribution of genome content similarity and microbe-microbe functional association indices

In order to evaluate the empirical properties of the indices described above, I calculate genome content similarity and microbe-microbe functional association metrics by comparing the genome of *Escherichia coli* str. K-12 substr. MG1655, *Clostridium tetani* E88, or *Halobacterium sp*. NRC-1, to the other 759 microbial genomes in STRING (S2A Fig). Only representative, distantly related, core genomes from STRING are included here. Accessory genomes, closely related to the three species in focus, are also included, but not ones related to core genomes other than *E*. *coli*, *C*. *tetani* or *Halobacterium sp*.

**Fig 2:**
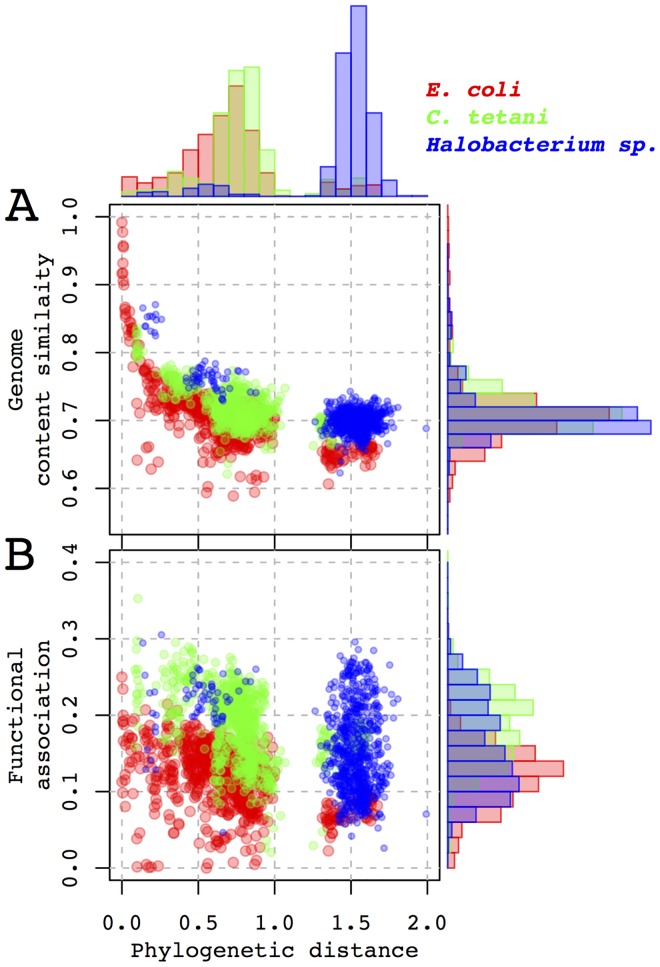
Relationship between new metrics and phylogenetic distances between the organisms. (A) Genome content similarity and (B) microbe-microbe functional association indices with phylogenetic distances between the species for three microbial taxa (shown on the top right) when compared to other core genomes from STRING and microbes related to the query genomes. Distribution of phylogenetic distances (in substitutions per site in 16S rRNA) is shown as histogram on the top, distributions of the indices are shown on the right of the corresponding plots.

Uneven representation of bacteria and archaea in STRING is evident from the bimodal distribution of phylogenetic distances between each of the three focal genomes and the rest of the included species (Fig 2 histograms on top), and from differences between distributions of phylogenetic distances measured from *Halobacterium* (archeae) and *E*. *coli* and *C*. *tetani* (bacteria).

Across all pairs of species examined, the two indices show a unimodal distribution ranging from 0.5 to 1 (Fig 2A) and 0 to 0.4 (Fig 2B) for genome content similarity and microbe-microbe functional association, respectively. Both indices decay with growth of phylogenetic distance. In the case of the genome content similarity index for *E*. *coli*, the relationship appears exponential, while in case of other genomes, they seem linear (Fig 2A). This can possibly be attributed to the presence of closely related strains of *E*. *coli* in STRING and the absence of a large number of closely related taxa for other genomes.

### Indices developed here are correlated with microbial co-occurrence in two ecological datasets

Correlation analysis is performed to reveal if new indices developed here can be useful in predicting microbial co-occurrence (S2B Fig). Pairwise genome content similarity, microbe-microbe functional association, and co-occurrence of the microbes are calculated for STRING genomes detected in following ecological datasets:

1. Co-occurrence between microbes from various habitats (marine and fresh water, soil, host-associated habitats and so on) is calculated from Operational Taxonomic Unit (OTU) and sample information in the Greengenes database, which includes 308 genomes.
2. Co-occurrence between microbial species from the human intestinal microbiome as reported in Levy and Borenstein, 2013 [21], which includes 127 genomes.

The two ecological datasets described above should represent different conditions for the metrics to predict associations between microbes within different habitats (dataset 1) or to capture concerted patterns of presence or absence of microbes within the same environment (dataset 2).

**Fig 3:**
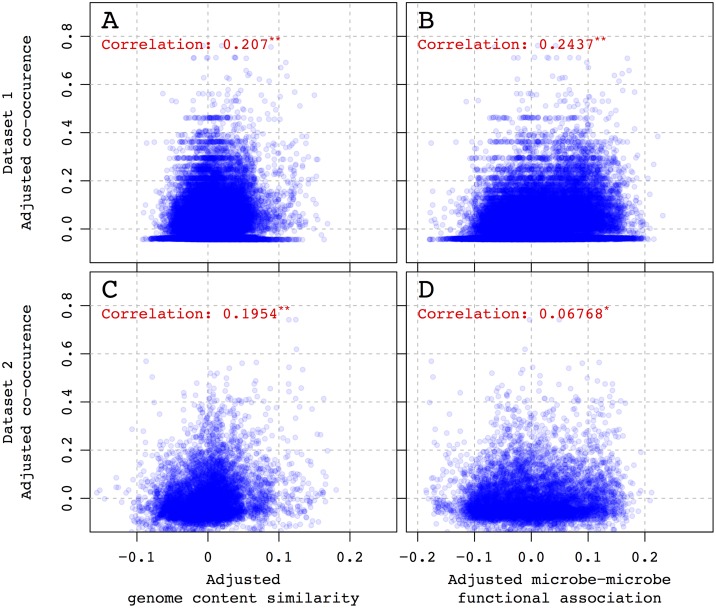
Relationship between co-occurrence and new metrics. Genome content similarity index (A) and (C) and microbe-microbe functional association index (B) and (D) in two different ecological datasets as shown in rows. In each plot, both response and independent variables are adjusted for phylogenetic distance between organisms. Pearson correlations are shown for every plot, “*” and “**” symbols denote associated Mantel p-value < 0.05 and < 0.01 respectively.

Next, I calculate partial correlations between the indices and co-occurrence accounting for phylogenetic relationships between the species for both ecological datasets (S2B Fig). Co-occurrence is correlated positively with the genome content similarity index in both ecological datasets (Fig 3), with Pearson correlations between the measures equal to 0.207 (p-value = 0.0001) in dataset 1 and 0.1954 (p-value = 0.0001) in dataset 2 (Fig 3A and 3C). This result means that the more similar the genomes of two microbes are, the more likely they are to be found together in the environment, thus highlighting the importance of habitat filtering in microbial community assembly both across the environments (dataset 1) and within similar ecological habitats (dataset 2).

Co-occurrence is also correlated positively with the microbe-microbe functional association index in both ecological datasets (Fig 3). The Pearson correlations between the measures are 0.2437 (p-value = 0.0001) and 0.06768 (p-value = 0. 0331) in datasets 1 and 2, respectively (Fig 3B and 3D). This result indicates that taxa, which tend to be found together, have higher potential for interaction at the molecular level as captured here through reconstructed protein-protein functional association network.

### Comparing indices developed here to previously existing methods using ecological data from human stool (dataset 2)

Ecological dataset 2 is also used to compare metrics developed here to previously reported metabolic competition and complementarity indices, which are constructed using KEGG pathways. To compare predictive power of different metrics, I perform Mantel regression analysis between co-occurrence as the response variable, and ether phylogenetic distance alone (regression model 1), or one of the four available genomics-based indices, two generated in this study and two existing ones, and phylogenetic distance between organisms (regression models 2 to 5), or all five predictors as the independent variables (regression model 6), in ecological datasets 2 (S2C Fig).

All of the tested regression models are statistically supported (Table 1), p-values associated with F-statistics are less than 0.05. Phylogenetic distances alone explain 9.84% of the variance in co-occurrence of microbes (regression 1). All of the genomic indices, when considered one at a time in a combination with phylogenetic distances between microbes also produce statistically supported models (p-values for t-statistics associated with coefficients for genomic indices is less than 0.05 in regressions 2 to 5) and explain a significant amount of the variation in the co-occurrence data (Table 1). Genome content similarity explains the highest fraction of the variance in addition to the fraction explained by phylogeny alone (3.44%), followed by the metabolic competition index, which accounts for 2.11% of the variance. Metabolic complementarity explains 1.7% of the variance in co-occurrence data, and the microbe-microbe functional association index explains less than 1% of the variance.

**Table 1:** Regressions analysis of co-occurrence using various genomics-based indices and phylogenetic distance.

Stats	Considered predictors	Regressions models of co-occurrence of microbes in ecological dataset 2					
		#1	#2	#3	#4	#5	#6
Estimated slope coefficient (t-statistic, p-value)	Genome content similarity		0.476 (17.53, 0.0001)				0.537 (11.84, 0.0001)
	Functional associations			0.093 (5.97, 0.0168)			-0.1 (-5.24, 0.0257)
	Metabolic competition				0.116 (13.63, 0.0001)		-0.013 (-0.88, 0.7098)
	Metabolic complementarity					-0.238 (-12.2, 0.0001)	-0.086 (-2.97, 0.1434)
	Phylogenetic distance	-0.15 (-29.08, 0.0001)	-0.13 (-24.93, 0.0001)	-0.148 (-28.72, 0.0001)	-0.135 (-25.75, 0.0001)	-0.143 (-27.85, 0.0001)	-0.128 (-24.52, 0.0001)
F-statistic (p-value)		846 (0.0001)	593 (0.0001)	442 (0.0001)	525 (0.0001)		245 (0.0001)
R^2^ (percent of variance in co-occurrence explained by used predictors)		9.84%	13.28%	10.25%	11.95%	11.54	13.65
R^2^ –R^2^ of regression #1 (percent of variance explained by genomic summary alone)			3.44%	0.41%	2.11%	1.7%	3.81%

Regression model 5, which combines all four genomics-based indices, does not seem to improve over the predictive ability of regression model 2, which includes phylogenetic distance and genome content similarity. Microbe-microbe functional association index is the only other significant predictor in model 6 (p-value 0.0257). The regression model 6 explains 3.81% of variance (in addition to phylogeny) marking the current predictive power of genomics-based techniques in predicting ecological associations between microbes.

### Similar genome content composition in a context of specific gene sets is detected in two co-occurring species of *Firmicutes*

**Fig 4:**
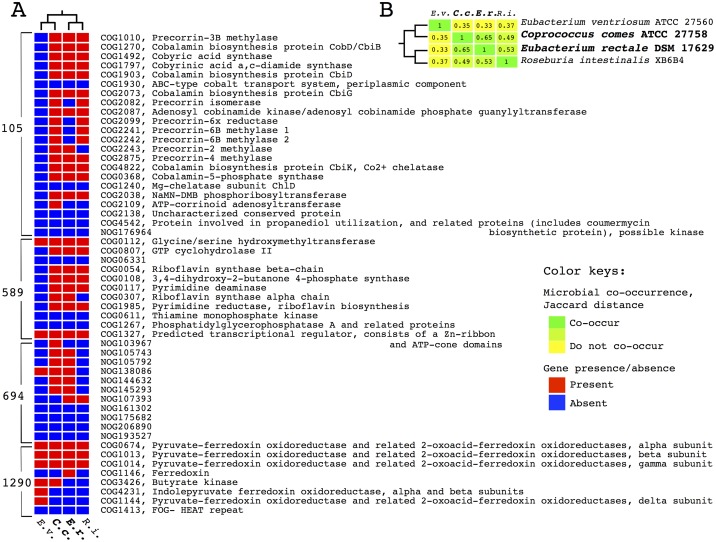
Putative pathways exhibiting similarity of gene content in two co-occurring lahnospiracea species. (A) Pattern of gene presence absence in two interacting species, *C*. *comes* and *E*. *rectale*, and their related species, *E*. *ventriosum* and *R*. *intestinalis*, in four identified gene sets of interest. Species name abbreviations are shown in the bottom of the heatmap, gene family annotations from STRING are shown on the right. Gene set IDs are on the left of the heatmap. (B) Patterns of co-occurrence of four species under consideration in human stool samples, ecological dataset 2, are shown as a heatmap. Official names of the organisms are shown on the right. Phylogenetic relationships between the species, as detected using 16S rRNA, are displayed as dendrogram on top and on the left in panes (A) and (B). Species name abbreviations at the bottom and top in panes (A) and (B). Names of co-occurring taxa are shown in bold. Color keys for both panels are on the right.

To evaluate the potential use of genomics-based methods developed here for understanding mechanisms driving microbe-microbe interaction I conduct analysis of putative pathways in one set of four taxa from phylum *Firmicutes*, found in stool samples. The set of taxa under consideration includes: *C*. *comes*, *E*. *rectale*, *R*. *intestinalis* and *E ventriosum* (Fig 4). Phylogenetic relationships between these four species inferred using 16S rRNA support *C*. *comes—E ventriosum; and E*. *rectale—R*. *intestinalis* as pairs of sister taxa. In this case, however, *C*. *comes*, *E*. *rectale* co-occurred more frequently (Jaccard index of 0.65) than other combinations of four taxa (Fig 4B).

Subsequently, I search for putative pathways exhibiting high enough level of genome content similarity between co-occurring taxa and sufficient overall pathway representation (see Materials and Methods section for details), in comparison to their not co-occurring sister lineages. Four gene sets are identified (Fig 4A). Three of those gene sets, according to the annotation, are related to riboflavin and cobalamin metabolism (gene sets 105 and 589) and energy metabolism (gene set 1290). Additionally, this search identifies one gene set which includes gene families of unknown function (gene set 694).

## Discussion

16S-based approaches to microbial ecology have been productive and have yielded useful discoveries [9,10,40], but at the same time they have several limitations: they do not provide a handle on mechanisms determining observed patterns, and are limited for communities which are hard to sample within ecological surveys. Genomics-based approaches to the problem might provide a handle on the mechanisms driving microbial community assembly and dynamics, complementing conclusions derived from ecological surveys. They might also help draw conclusions for microbes from poorly sampled communities. However such approaches have been limited so far and only two metrics, metabolic competition and complementarity indices, exist [21,41].

### Major findings of the study and their implications

In this study, I attempt to advance genomics-based methods for understanding ecological associations between microbes. I introduce two novel genome-wide measures of microbe-microbe interaction—genome content similarity and microbe-microbe functional association indices—and demonstrate how these measures predict associations between microbes in different environments. Specifically, I show that both metrics predict common environmental affiliations of bacterial species when ecological divergence between habitats is high (Fig 3, dataset 1). The predictive power of both indices stays significant even when the surveyed environmental conditions become more similar and the expected ecological differentiation between habitats is reduced (Fig 3, dataset 2). This indicates the presence of detectable genome-wide signal of co-occurrence of the microbes in both highly differentiated and similar environments.

Regression analysis also allows me to compare indices proposed here to the previously proposed metrics. The results indicate that genome content similarity index explains patterns of microbial co-occurrence better than sophisticated metabolic competition index constructed upon KEGG pathway annotation (3.4% versus 2.1% of variance explained, Table 1, regression models 2 and 4 respectively).

While it is clear that genomic information is one of the major factors determining species ecology, it is still not known to what extent ecological interactions between the species, as measured here by co-occurrence, can be explained by genomic data. In this study I aim to address this question. Using regression analysis, I show that genomic summaries alone predict co-occurrence of microbes even when accounting for phylogenetic relationships between the organisms and explain up to 4% of the variance in co-occurrence data (Table 1, regression model 6).

This study also finds phylogenetic relatedness of the organisms to be the best predictor of their co-occurrence. On it’s own phylogenetic relatedness explains about 10% of the variance in co-occurrence data. This findings highlight the importance of the evolutionary process in the emergence of ecologically important traits in microbial genomes and in agreement with previous reports [9,10]. The observation, however, contradicts “limiting similarity hypothesis” in community ecology [42]. Empirical studies suggesting closely related species tend to exclude each other have been reported [43,44] but contradictory reports also have been published [45,46]. Existence of evidence pointing in different directions might be an indication that the effect of “limiting similarity” can only be detected for specific values of divergence, or specific time scales on which a community is surveyed, it might also vary depending on the rate of evolution of the traits important in particular ecosystem. Elucidating these possibilities would require in depth analysis and is not pursued in the study.

It is important to note that the indices introduced here are informed only by the genome content and structure of various microbial species and not by biochemical annotation of proteins. While some biases in the resolution of the protein functional association networks predicted using genome content are expected, given that taxa are not sampled into genome sequencing studies at random [47], they should nevertheless be small in comparison to biases in experimental biochemical annotation. Therefore, the metrics introduced here should be more reliably applicable to a wide range of microbes, not only the well-studied taxa with large number of annotated metabolic pathways.

The positive correlation between co-occurrence as measured by 16S rRNA and genome content similarity detected here (Table 1) highlights the importance of habitat filtering processes in community assembly [48,49]. This finding does not exclude other processes, such as species assortment, as important drivers of community assembly [50]. Perhaps processes that result in differences in genome composition, cooperation or cheating [15,51,52], operate on the level of a small set of biological functions and go undetected at the level of the genome. For instance, the loss of siderophore biosynthesis genes, but not reuptake genes, by some strains of marine *Vibrionaceae* leads to differences in genome content composition in co-existing strains but only for one particular cluster of functionally linked genes, not genome-wide [14].

### Potential limitations of proposed methods

Additionally, similarities observed at the level of genome composition do not necessarily translate into similarities at the level of mRNA or protein expression. It has been shown that social cheating in *Pseudomonas* quorum sensing arises from changes in gene expression rather than complete loss of the genetic modules encoding quorum-controlled factors [51]. Quick loss of metabolic independence due to loss-of-function mutations in protein coding sequences, but not loss of detectable orthologs, are also known [53]. The methods introduced here assume that all the proteins present in the genomes of a microbial species are expressed and functional. This unrealistic assumption, in theory, could be relaxed, but such a development would require information on genome-wide patterns of gene expression for both species in question, grown under the same conditions. This kind of information is limited for reference genomes but should be accessible for wild strains from metatranscriptomics studies [54].

Avoiding the use of biochemical pathway annotation is advantageous, as it allows for the incorporation of signals from large numbers of proteins (S1 Fig). On the other hand, using genomics-based predictions about protein functional associations makes it harder to interpret the results, especially for the microbe-microbe functional association index, as “functional association” is broadly defined here and encompasses an ensemble of interactions ranging form direct physical contact to genetic regulation to involvement in the same biochemical process [24].

It should be highlighted that both genome content similarity and the microbe-microbe functional association index are based on static gene family annotations from STRING, which assumes that all the genes from the same automatically predicted orthologous group have same functional associations. This is clearly a naïve assumption, given that proteins evolve new functions across phylogeny [55,56]. For instance, comparative genomics study on the SecA_DEAD domain protein in some Gram-negative microbes suggested several functional associations for some of the proteins from the SecA family (COG0653) from STRING, but not all of them [57]. Therefore, investigating the role of lineage specific protein evolution on this type of inference could be of interest.

### Gene set specific analysis

Genome content similarity and microbe-microbe functional association indices summarize information genome-wide. In reality, however, only a fraction of the genome might be mediating ecological interactions. Therefore, one potential extension of the methods introduced here is to predict small set of putative pathways driving ecological interactions between microbes. Experimental and computational detection of protein-protein interactions in host-microbe systems [58,59] allowed to discover a number of microbial proteins potentially interacting with human proteins. Detailed analysis of protein-protein functional association networks in search of clusters of gene families contributing to the elevated microbe-microbe functional association index could lead to the discovery of some promising candidate molecular systems. In the case of genome content similarity, one approach is to search for gene sets exhibiting higher than expected compositional similarity. In this manuscript, such a survey was conducted for two co-occurring species from phylum *Firmicutes*, family *Lahnospiracea*.

Several promising candidate gene sets were discovered (gene sets 105, 589, 694 and 1290). Three of the gene sets have assigned metabolic functions (105, 589 and 1290). For instance, gene set 1290 includes genes linked to butyrate metabolism. Sporadic phylogenetic distribution of butyric acid producing enzymes, potentially driven by HGT, has been reported in *Lahnospiracea* [60]. Here the evidence indicates that lahnospiracea species with similar set of butyric acid metabolism related genes, *C*. *comes* and *E*. *rectale*, also tend to co-occur in the environment. Gene set 694 containes genes of unknown function, which could not have been identified by KEGG pathways analysis.

Gene set 105 includes genes related to vitamin B12 biosynthesis and while the overall gene family profile in this gene set is similar in co-occurring *C*. *comes* and *E*. *rectale*, several genes catalyzing initial steps in the pathways [61–63] are missing from *E*. *rectale*. These findings potentially suggest exchange of intermediates of vitamin B12 biosynthesis between co-occurring *E*. *rectale* and *C*. *comes*. Overall, the gene sets discovered here constitute a list of promising potential candidates for further functional studies but at this point inspire speculation.

Evolutionary processes generating detected patterns are not evaluated here and would require more in-depth phylogenetic analysis. However, a parsimonious assessment of the observed gene presence and absence profiles in the examined four taxa (Fig 4A) suggests that identification of the gene sets might be attributed to gene loss in *E*. *ventriosum*, related to *C*. *comes*, for two gene sets (105 two 589) and concerted gene gain and loss by two co-occurring taxa (gene set 694 and 1290 respectively).

### Conclusions

In summary, this study finds phylogenetic relatedness to be strongest predictor of microbial co-occurrence (explains about 10% of the variance in microbial co-occurrence). Genome content similarity index is also identified as a strong predictor (explains 3.5% of the variance), highlighting the importance of habitat filtering in microbial community assembly. Genome content similarity index provides an improvement over more sophisticated metabolic competition index which requires metabolic pathway annotation for each of the genomes and is highly limited for poorly studied microbes. Despite the fact that none-trivial fraction of variance in co-occurrence data is explained by genomic indices, detected explanatory power is rather modest. This highlights the need for the development of methods to improve current genomic techniques to help in understanding the inner workings of microbial communities.

## Materials and methods

Main functionalities and key data required for the analysis carried out in this study have been included within genomics2ecology R package deposited to GitHub [64]. Scripts and data necessary to generate results of this paper are available via another GitHub repository [65].

### Genomes, genes, gene families and functional associations between gene families

The files species.v10.txt, species.mappings.v10.txt and COG.mappings.v10.txt, which provide information on species and genome annotation, relationships between genomes, genes and gene families, were downloaded from the STRING version 10.0 website [66]. The file COG.links.detailed.v10.txt, which provides information on functional associations between gene families, was also downloaded from the database website. Information was extracted from these files using custom Python scripts.

### Network of functionally linked gene families

The global network of all the gene families existing in the STRING database was defined as a collection of all nodes (orthologous groups from STRING) and all edges (gene neighbor scores from STRING above critical value) connecting the nodes. The network is available within the genomics2ecology R package under the reference_network table.

Gene neighbor score values were not derived within this study but obtained from COG.links.detailed.v10.txt file from STRING [66]. A critical score value of 275 was used to define if link between two gene families exists of not. The critical value of 275 was chosen because it corresponds to the best values of both specificity and sensitivity in the ROC curve [57] when the scores are evaluated on a set of known functionally related proteins.

To compute microbe-microbe functional association index values edges of reference gene network were treated as unweighted.

### Putative pathways and complexes

The genome content similarity index was calculated based on sets of functionally linked genes (putative pathways and complexes). To identify such gene sets the global network of gene families, constructed as described above, was clustered with mcl-14-137 [67,68]. Edge weights (gene neighbor score values above 275) in the global network of gene families were unit-based normalized by subtracting minimal weight (275) from each value and then dividing the result by the range (1000–275). This weight adjustment scheme is similar to what is recommended in the literature in analysis of other networks with mcl [69]. The inflation value for mcl was set to 4 to obtain fine-grained clusters, and the program was run in --abc mode to accommodate the format of input data, for the rest of parameters default settings were employed. The obtained clusters of gene families were further treated as putative protein pathways and complexes. The gene sets are available within genomics2ecology R package under reference_gene_sets data structure.

Given that I further used putative protein pathways and complexes to derive the genome content similarity index, it was of interest to understand how the fraction of genes contributing to putative protein pathways and complexes varies between genomes. I calculated this percentage for 308 genomes using clusters from mcl and protein.aliases.v10.txt file from STRING. I also obtained information on the percent of ORFs in KEGG pathways for the same genomes from JGI.

### New metrics for quantifying associations between microbes

#### Genome content similarity index

This index was calculated as illustrated in Fig 1A. For every putative pathway network (see section above for details on how putative pathways are obtained), which includes at least 4 gene families, presence and absence of every gene family in each of the genomes is obtained from STRING. A similarity score was calculated for every gene set as the number of gene families in the set with the same presence/absence states in two genomes. This number was normalized by the size of the gene set to avoid large sets driving the inference. In the last step, the similarity scores associated with different clusters were averaged. Only gene sets present in at least one of the genomes were included to avoid inflation of the index due to gene sets absent from both organisms. Presence of at least 5% of the gene families from the putative pathway in a given genome was considered as indication of a pathway’s presence.

#### Microbe-microbe functional association index

This index was calculated for every relevant pair of genomes as illustrated in Fig 1B. A protein-protein functional association network for a given pair of organisms was derived from the reference network (see section above for details on how reference network is obtained) by including gene families, nodes of the network, present in at least one of the genomes under consideration and links connecting those gene families, edges of the network, (Fig 1B). Gene families were also labeled to denote if a protein from a given gene family was present in genome A, genome B, or both, producing 3 types of gene families (A, B and both labels, represented by blue, yellow and green colors, in Fig 1B). Such a network contains 6 types of undirected edges, connecting two nodes of 3 different types to each other (both to A, both to B, both to both, A to A, A to B and B to B).

Network edges, which connect proteins encoded exclusively in two different genomes (A to B edges in Fig 1B), would have to cross genome boundaries in order to exist in the system (Fig 1B). Other edges can be formed within one genome. Therefore, I defined the microbe-microbe functional association index between two microbes as a fraction of edges which would have to cross organismal boundaries among all the edges connecting gene families encoded exclusively in one of the genomes (Fig 1B).

### Mapping STRING genomes to two ecological datasets

In this study, I used two ecological datasets to understand whether genomics-based indices can predict co-occurrence of microbes in the environment. The first dataset was from the Greengenes database files from May 2013 [70,71], and the second one was from a previously published study [21].

To map STRING genomes onto the Greengenes OTUs, I first obtained the 16S rRNA sequences for 1780 reference genomes (STRING_16S_tid.fa file in the GitHub repository [65]) from the Ribosomal Database Project [72] by matching the NCBI taxonomy ID provided within the STRING database and in the files current_Archaea_unaligned.gb and current_Bacteria_unaligned.gb files downloaded from the RDP website in June 2015. Sequences were extracted from current_Archaea_unaligned.fa and current_Bacteria_unaligned.fa. Taxa not found in RDP were found in IMG JGI [73]. The longest 16S rRNA sequence for each genome was selected.

Data from the Greengens database were handled as follows: one representative sequence which had “isolation source”, “authors” and “title” annotation in its arb record was selected for each of the 97% OTUs from Greengens (97_otu_map.txt file from gg_13_5_otus.tar.gz archive). Sequences shorter than 900 and 1,200 nucleotides in the case of Archaea and Bacteria, respectively, were removed. The rest of the sequences were searched with 16S rRNAs from STRING genomes using blastn. Weak blast hits with less than 95% identity or covering less than 85% of the query sequence length were ignored. 16S rRNAs from STRING genomes and corresponding Greengenes sequence which produces strong blast hits were collected. Collected sequences were aligned to each other using the bacterial 16S model and clustered using complete linkage clustering based on sequence identity with tools available on the RDP website, 97% identity cut-off was used for clustering. One Greengenes OTU and one STRING genome were selected to represent each cluster that contained both STRING genome and Greengenes OTU. In total 1119 STRING genomes were assigned to Greengens OTU this way. R code is in get_data_ds1.R.txt on GitHub [65].

In order to establish correspondence between 154 genomes from [21] (file sd01.xlsx from PNAS website, sheet A, row names) and STRING genomes, I first modified the “official name” of the STRING genomes by replacing space, dot or dash characters with underscore characters, then replacing repeated underscores with just one, and the looked for exact match between modified STRING genome names and genome names from [21]. Using this method way 83 genomes from [21] were matched. The rest of the genomes were assigned manually, by strain if possible, otherwise by species name. If several strains of the same species were present in STRING, one strain was selected at random to represent the genome in subsequent analysis. If no species with the same name was present in STRING, the genome was excluded from the dataset. A total of 127 genomes were included into dataset 2. The R code is in get_data_ds2.R, the list of assigned genomes is in genomes_ds2.txt file in GitHub [65].

### Co-occurrence between Greengenes OTUs

Sample information was extracted from gg_13_5_arb_records files obtained from the Greengenes ftp site [71]. Record files link sequences to samples. Only sequences annotated with “isolation source”, “authors” and “title” were used. Sample IDs were created by concatenating “isolation source”, “authors” and “title” fields. Individual 16S rRNA sequences in Greengenes are grouped into OTUs. 97% identity OTUs were utilized here (97_otu_map.txt). OTUs which did not match STRING genomes, OTUs present in less than 3 samples and samples with less than 3 OTUs were removed. In total 308 OTUs (out of 1119 initially matched to genome from STRING) and 532 samples were retained for further analysis. A similar strategy for OTU/sample filtering was used before [10]. The co-occurrence between the OTUs was calculated as Jaccard similarity coefficients [74] between profiles of OTU presence/absence in samples as was previously done in [21]. R code is in get_data_ds2.R; the generated matrix of species co-occurrence is in cooccurence_ds1.txt, and a list of assigned genomes is in genomes_ds1.txt on GitHub [65].

### Co-occurrence data and genomics-based indices from [21]

Metabolic complementarity and competition indices (file sd01.xlsx, sheet A) as well as co-occurrence measures using the Jaccard similarity coefficient (file sd01.xlsx, sheet B) for human gut microbiome data are provided as part of supporting information for [21] and were downloaded from the PNAS website, competition_ds2_full.txt and cooccurrence_ds2.txt on GitHub [65].

### Genome content similarity and microbe-microbe functional association indices

Genome content similarity and microbe-microbe functional association indices were calculated for every relevant pair of genomes (as described in Results section) using similarity and functional_association functions from the genomics2ecology R package [64]. Code is provided in get_data_ds1.R and get_data_ds2.R files; generated genomics based indices are in similarities_ds1.txt, similarities_ds2.txt, similarities_F2.txt, associations_ds1.txt, associations_ds2.txt, associations_F2.txt files on GitHub [65].

### Phylogenetic trees

To approximate relationships between species in two ecological datasets and for the collection of species used to create Fig 2, I first aligned 16S rRNA sequences from relevant STRING genomes using the RDP web-server [72]. I then reconstructed 16S rRNA phylogeny using FastTree 2.1.9 [75], files STRING_16S_ds1_FastTree, STRING_16S_ds2_FastTree, STRING_16S_fig2_FastTree on GitHub [65]. FastTree was compiled for double precision to improve length estimation of very short branches. It is necessary to note that the procedure adopted here is not intended to recover a precise species tree but rather to account for a strong signal of ancestry between closely related species.

### Correlation between genomics-based indices and co-occurrence in environmental samples and regression analysis

To address how genomics-based indices are related to co-occurrence of species in environmental samples I used partial Mantel test accounting for phylogenetic distance between species [76]. S2B Fig provides a graphical guide of the process. To calculate partial correlations raw species phylogeny, data on co-occurrence, genome content similarity and microbe-microbe functional association indices were used for ecological dataset 1 and 2. For dataset 2 metabolic competition and complementarity indices were also used. Phylogenetic distances between the species in each of the trees was calculated from the phylogenetic tree from the corresponding dataset using the cophenetic function from the ape R package version 3.4 [77]. The tests were performed using the vegan R package version 2.3–5 [78]. Adjustment for phylogenetic distance was done because genomes cannot be considered as independent observations as they are related to each other through evolutionary processes. The code is in analysis.R on GitHub [65].

In addition to partial correlation, I performed Mantel regression analysis of co-occurrence of microbes in the environment and genomics-indices in ecological dataset 2 (See S2C Fig for graphical guide). This analysis was performed using phytools R package version 0.5–20 [79] on the same set of raw data as used in the correlation analysis (analysis.R on GitHub [65]).

### Analysis of individual gene sets

To identify gene sets potential driving co-occurrence of *C*. *comes* and *E*. *rectale* I first identified gene sets that included at least 8 gene families, showed overall similarity of at least 0.6, and overall gene set representation of at least 0.6 when co-occurring *C*. *comes* and *E*. *rectale* were compared to each. Then I excluded from this list gene sets which were also identified when *R*. *intestinalis* to *E ventriosum* in the same way. Resulting data were visualized using gplots R package.

## Supporting information

S1 Fig Percent of ORFs in a KEGG pathways and b predicted pathways of different size. (A) Percent of the ORFs included in a KEGG pathways or (B) putative pathways predicted with MCL in 308 genomes from STRING from ecological dataset 1 which is introduced later in the paper.
(PDF)

S2 Fig Analysis workflow. Graphical representation of statistical analysis workflow.
(PDF)

## References

[1] Brogden KA, Guthmiller JM, Taylor CE. Human polymicrobial infections. Lancet Lond Engl. 2005;365: 253–255. 10.1016/S0140-6736(05)17745-9 PMC7119324 15652608

[2] Rohlke F, Stollman N. Fecal microbiota transplantation in relapsing Clostridium difficile infection. Ther Adv Gastroenterol. 2012;5: 403–420. 10.1177/1756283X12453637 PMC3491681 23152734

[3] Roos K, Håkansson EG, Holm S. Effect of recolonisation with “interfering” alpha streptococci on recurrences of acute and secretory otitis media in children: randomised placebo controlled trial. BMJ. 2001;322: 210–212. 11159619 10.1136/bmj.322.7280.210 PMC26587

[4] Di Pierro F, Donato G, Fomia F, Adami T, Careddu D, Cassandro C, et al Preliminary pediatric clinical evaluation of the oral probiotic Streptococcus salivarius K12 in preventing recurrent pharyngitis and/or tonsillitis caused by Streptococcus pyogenes and recurrent acute otitis media. Int J Gen Med. 2012;5: 991–997. 10.2147/IJGM.S38859 23233809 PMC3516470

[5] Moralejo-Gárate H, Mar’atusalihat E, Kleerebezem R, van Loosdrecht MCM. Microbial community engineering for biopolymer production from glycerol. Appl Microbiol Biotechnol. 2011;92: 631–639. 10.1007/s00253-011-3359-3 21674168

[6] Großkopf T, Soyer OS. Synthetic microbial communities. Curr Opin Microbiol. 2014;18: 72–77. 10.1016/j.mib.2014.02.002 24632350 PMC4005913

[7] Woese CR, Fox GE. Phylogenetic structure of the prokaryotic domain: The primary kingdoms. Proc Natl Acad Sci. 1977;74: 5088–5090. 270744 10.1073/pnas.74.11.5088 PMC432104

[8] Case RJ, Boucher Y, Dahllöf I, Holmström C, Doolittle WF, Kjelleberg S. Use of 16S rRNA and rpoB genes as molecular markers for microbial ecology studies. Appl Environ Microbiol. 2007;73: 278–288. 10.1128/AEM.01177-06 17071787 PMC1797146

[9] Faust K, Sathirapongsasuti JF, Izard J, Segata N, Gevers D, Raes J, et al Microbial Co-occurrence Relationships in the Human Microbiome. PLoS Comput Biol. 2012;8: e1002606 10.1371/journal.pcbi.1002606 22807668 PMC3395616

[10] Chaffron S, Rehrauer H, Pernthaler J, von Mering C. A global network of coexisting microbes from environmental and whole-genome sequence data. Genome Res. 2010;20: 947–959. 10.1101/gr.104521.109 20458099 PMC2892096

[11] Barberán A, Bates ST, Casamayor EO, Fierer N. Using network analysis to explore co-occurrence patterns in soil microbial communities. ISME J. 2012;6: 343–351. 10.1038/ismej.2011.119 21900968 PMC3260507

[12] Steele JA, Countway PD, Xia L, Vigil PD, Beman JM, Kim DY, et al Marine bacterial, archaeal and protistan association networks reveal ecological linkages. ISME J. 2011;5: 1414–1425. 10.1038/ismej.2011.24 21430787 PMC3160682

[13] Eiler A, Heinrich F, Bertilsson S. Coherent dynamics and association networks among lake bacterioplankton taxa. ISME J. 2012;6: 330–342. 10.1038/ismej.2011.113 21881616 PMC3260505

[14] Cordero OX, Ventouras L-A, DeLong EF, Polz MF. Public good dynamics drive evolution of iron acquisition strategies in natural bacterioplankton populations. Proc Natl Acad Sci. 2012;109: 20059–20064. 10.1073/pnas.1213344109 23169633 PMC3523850

[15] Van Leuven JT, Meister RC, Simon C, McCutcheon JP. Sympatric Speciation in a Bacterial Endosymbiont Results in Two Genomes with the Functionality of One. Cell. 2014;158: 1270–1280. 10.1016/j.cell.2014.07.047 25175626

[16] Dehority BA. Effects of microbial synergism on fibre digestion in the rumen. Proc Nutr Soc. 1991;50: 149–159. 1661009 10.1079/pns19910026

[17] Cordero OX, Wildschutte H, Kirkup B, Proehl S, Ngo L, Hussain F, et al Ecological Populations of Bacteria Act as Socially Cohesive Units of Antibiotic Production and Resistance. Science. 2012;337: 1228–1231. 10.1126/science.1219385 22955834

[18] Ng W-L, Bassler BL. Bacterial quorum-sensing network architectures. Annu Rev Genet. 2009;43: 197–222. 10.1146/annurev-genet-102108-134304 19686078 PMC4313539

[19] Li YF, Costello JC, Holloway AK, Hahn MW. “reverse Ecology” and the Power of Population Genomics. Evolution. 2008;62: 2984–2994. 10.1111/j.1558-5646.2008.00486.x 18752601 PMC2626434

[20] Borenstein E, Feldman MW. Topological signatures of species interactions in metabolic networks. J Comput Biol J Comput Mol Cell Biol. 2009;16: 191–200. 10.1089/cmb.2008.06TT PMC3035845 19178139

[21] Levy R, Borenstein E. Metabolic modeling of species interaction in the human microbiome elucidates community-level assembly rules. Proc Natl Acad Sci U S A. 2013;110: 12804–12809. 10.1073/pnas.1300926110 23858463 PMC3732988

[22] Carr R, Borenstein E. NetSeed: a network-based reverse-ecology tool for calculating the metabolic interface of an organism with its environment. Bioinformatics. 2012;28: 734–735. 10.1093/bioinformatics/btr721 22219204

[23] Steinway SN, Biggs MB, L TP Jr, Papin JA, Albert R. Inference of network dynamics and metabolic interactions in the gut microbiome. PLOS Comput Biol. 2015;11: e1004338 10.1371/journal.pcbi.1004338 26102287 PMC4478025

[24] Bowers PM, Pellegrini M, Thompson MJ, Fierro J, Yeates TO, Eisenberg D. Prolinks: a database of protein functional linkages derived from coevolution. Genome Biol. 2004;5: R35 10.1186/gb-2004-5-5-r35 15128449 PMC416471

[25] Sait M, Kamneva OK, Fay DS, Kirienko NV, Polek J, Shirasu-Hiza MM, et al Genomic and Experimental Evidence Suggests that Verrucomicrobium spinosum Interacts with Eukaryotes. Front Microbiol. 2011;2. 10.3389/fmicb.2011.00211 PMC3196152 22022322

[26] Kolesov G, Wunderlich Z, Laikova ON, Gelfand MS, Mirny LA. How gene order is influenced by the biophysics of transcription regulation. Proc Natl Acad Sci U S A. 2007;104: 13948–13953. 10.1073/pnas.0700672104 17709750 PMC1955771

[27] Ferrer L, Dale JM, Karp PD. A systematic study of genome context methods: calibration, normalization and combination. BMC Bioinformatics. 2010;11: 493 10.1186/1471-2105-11-493 20920312 PMC3247869

[28] Ferrer L, Shearer AG, Karp PD. Discovering novel subsystems using comparative genomics. Bioinforma Oxf Engl. 2011;27: 2478–2485. 10.1093/bioinformatics/btr428 PMC3167049 21775308

[29] Kamneva OK, Knight SJ, Liberles DA, Ward NL. Analysis of genome content evolution in PVC bacterial super-phylum: assessment of candidate genes associated with cellular organization and lifestyle. Genome Biol Evol. 2012;4: 1375–1390. 10.1093/gbe/evs113 23221607 PMC3542564

[30] Cimermancic P, Medema MH, Claesen J, Kurita K, Wieland Brown LC, Mavrommatis K, et al Insights into Secondary Metabolism from a Global Analysis of Prokaryotic Biosynthetic Gene Clusters. Cell. 2014;158: 412–421. 10.1016/j.cell.2014.06.034 25036635 PMC4123684

[31] Chen L-H, Köseoğlu VK, Güvener ZT, Myers-Morales T, Reed JM, D’Orazio SEF, et al Cyclic di-GMP-dependent Signaling Pathways in the Pathogenic Firmicute Listeria monocytogenes. PLoS Pathog. 2014;10. 10.1371/journal.ppat.1004301 PMC4125290 25101646

[32] Haft DH, Selengut J, Mongodin EF, Nelson KE. A guild of 45 CRISPR-associated (Cas) protein families and multiple CRISPR/Cas subtypes exist in prokaryotic genomes. PLoS Comput Biol. 2005;1: e60 10.1371/journal.pcbi.0010060 16292354 PMC1282333

[33] Kalyuzhnaya MG, Korotkova N, Crowther G, Marx CJ, Lidstrom ME, Chistoserdova L. Analysis of Gene Islands Involved in Methanopterin-Linked C1 Transfer Reactions Reveals New Functions and Provides Evolutionary Insights. J Bacteriol. 2005;187: 4607–4614. 10.1128/JB.187.13.4607-4614.2005 15968072 PMC1151760

[34] Köseoğlu VK, Heiss C, Azadi P, Topchiy E, Güvener ZT, Lehmann TE, et al Listeria monocytogenes exopolysaccharide: origin, structure, biosynthetic machinery and c-di-GMP-dependent regulation. Mol Microbiol. 2015;96: 728–743. 10.1111/mmi.12966 25662512

[35] Bäckhed F, Ley RE, Sonnenburg JL, Peterson DA, Gordon JI. Host-bacterial mutualism in the human intestine. Science. 2005;307: 1915–1920. 10.1126/science.1104816 15790844

[36] Konopka A. What is microbial community ecology? ISME J. 2009;3: 1223–1230. 10.1038/ismej.2009.88 19657372

[37] Rosenzweig RF, Sharp RR, Treves DS, Adams J. Microbial evolution in a simple unstructured environment: genetic differentiation in Escherichia coli. Genetics. 1994;137: 903–917. 7982572 10.1093/genetics/137.4.903 PMC1206068

[38] González-Pastor JE. Cannibalism: a social behavior in sporulating Bacillus subtilis. FEMS Microbiol Rev. 2011;35: 415–424. 10.1111/j.1574-6976.2010.00253.x 20955377

[39] Arnosti C. Microbial extracellular enzymes and the marine carbon cycle. Annu Rev Mar Sci. 2011;3: 401–425. 10.1146/annurev-marine-120709-142731 21329211

[40] Duran-Pinedo AE, Paster B, Teles R, Frias-Lopez J. Correlation Network Analysis Applied to Complex Biofilm Communities. PLoS ONE. 2011;6: e28438 10.1371/journal.pone.0028438 22163302 PMC3233593

[41] Zelezniak A, Andrejev S, Ponomarova O, Mende DR, Bork P, Patil KR. Metabolic dependencies drive species co-occurrence in diverse microbial communities. Proc Natl Acad Sci. 2015;112: 6449–6454. 10.1073/pnas.1421834112 25941371 PMC4443341

[42] Abrams P. The Theory of Limiting Similarity. Annu Rev Ecol Syst. 1983;14: 359–376.

[43] Peay KG, Belisle M, Fukami T. Phylogenetic relatedness predicts priority effects in nectar yeast communities. Proc Biol Sci. 2012;279: 749–758. 10.1098/rspb.2011.1230 21775330 PMC3248732

[44] Violle C, Nemergut DR, Pu Z, Jiang L. Phylogenetic limiting similarity and competitive exclusion. Ecol Lett. 2011;14: 782–787. 10.1111/j.1461-0248.2011.01644.x 21672121

[45] Fritschie KJ, Cardinale BJ, Alexandrou MA, Oakley TH. Evolutionary history and the strength of species interactions: testing the phylogenetic limiting similarity hypothesis. Ecology. 2014;95: 1407–1417. 25000771 10.1890/13-0986.1

[46] Mouillot D, Simková A, Morand S, Poulin R. Parasite species coexistence and limiting similarity: a multiscale look at phylogenetic, functional and reproductive distances. Oecologia. 2005;146: 269–278. 10.1007/s00442-005-0194-1 16049715

[47] Wu D, Hugenholtz P, Mavromatis K, Pukall R, Dalin E, Ivanova NN, et al A phylogeny-driven genomic encyclopaedia of Bacteria and Archaea. Nature. 2009;462: 1056–1060. 10.1038/nature08656 20033048 PMC3073058

[48] Keddy PA. Assembly and response rules: two goals for predictive community ecology. J Veg Sci. 1992;3: 157–164.

[49] Borenstein E, Kupiec M, Feldman MW, Ruppin E. Large-scale reconstruction and phylogenetic analysis of metabolic environments. Proc Natl Acad Sci. 2008;105: 14482–14487. 10.1073/pnas.0806162105 18787117 PMC2567166

[50] Cody ML, Diamond JM. Ecology and Evolution of Communities. Harvard University Press; 1975.

[51] Sandoz KM, Mitzimberg SM, Schuster M. Social cheating in Pseudomonas aeruginosa quorum sensing. Proc Natl Acad Sci. 2007;104: 15876–15881. 10.1073/pnas.0705653104 17898171 PMC2000394

[52] Pfeiffer T, Bonhoeffer S. Evolution of cross-feeding in microbial populations. Am Nat. 2004;163: E126–135. 10.1086/383593 15266392

[53] Hillesland KL, Lim S, Flowers JJ, Turkarslan S, Pinel N, Zane GM, et al Erosion of functional independence early in the evolution of a microbial mutualism. Proc Natl Acad Sci U S A. 2014;111: 14822–14827. 10.1073/pnas.1407986111 25267659 PMC4205623

[54] Yost S, Duran-Pinedo AE, Teles R, Krishnan K, Frias-Lopez J. Functional signatures of oral dysbiosis during periodontitis progression revealed by microbial metatranscriptome analysis. Genome Med. 2015;7: 27 10.1186/s13073-015-0153-3 25918553 PMC4410737

[55] Soskine M, Tawfik DS. Mutational effects and the evolution of new protein functions. Nat Rev Genet. 2010;11: 572–582. 10.1038/nrg2808 20634811

[56] Liberles DA, Teichmann SA, Bahar I, Bastolla U, Bloom J, Bornberg-Bauer E, et al The interface of protein structure, protein biophysics, and molecular evolution. Protein Sci Publ Protein Soc. 2012;21: 769–785. 10.1002/pro.2071 PMC3403413 22528593

[57] Kamneva OK, Poudel S, Ward NL. Proteins Related to the Type I Secretion System Are Associated with Secondary SecA_DEAD Domain Proteins in Some Species of Planctomycetes, Verrucomicrobia, Proteobacteria, Nitrospirae and Chlorobi. PLoS ONE. 2015;10: e0129066 10.1371/journal.pone.0129066 26030905 PMC4452313

[58] Huo T, Liu W, Guo Y, Yang C, Lin J, Rao Z. Prediction of host—pathogen protein interactions between Mycobacterium tuberculosis and Homo sapiens using sequence motifs. BMC Bioinformatics. 2015;16: 100 10.1186/s12859-015-0535-y 25887594 PMC4456996

[59] Blasche S, Arens S, Ceol A, Siszler G, Schmidt MA, Häuser R, et al The EHEC-host interactome reveals novel targets for the translocated intimin receptor. Sci Rep. 2014;4: 7531 10.1038/srep07531 25519916 PMC4269881

[60] Meehan CJ, Beiko RG. A Phylogenomic View of Ecological Specialization in the Lachnospiraceae, a Family of Digestive Tract-Associated Bacteria. Genome Biol Evol. 2014;6: 703–713. 10.1093/gbe/evu050 24625961 PMC3971600

[61] Blanche F, Thibaut D, Famechon A, Debussche L, Cameron B, Crouzet J. Precorrin-6x reductase from Pseudomonas denitrificans: purification and characterization of the enzyme and identification of the structural gene. J Bacteriol. 1992;174: 1036–1042. 1732193 10.1128/jb.174.3.1036-1042.1992 PMC206185

[62] Thibaut D, Couder M, Famechon A, Debussche L, Cameron B, Crouzet J, et al The final step in the biosynthesis of hydrogenobyrinic acid is catalyzed by the cobH gene product with precorrin-8x as the substrate. J Bacteriol. 1992;174: 1043–1049. 1732194 10.1128/jb.174.3.1043-1049.1992 PMC206186

[63] Roth JR, Lawrence JG, Rubenfield M, Kieffer-Higgins S, Church GM. Characterization of the cobalamin (vitamin B12) biosynthetic genes of Salmonella typhimurium. J Bacteriol. 1993;175: 3303–3316. 8501034 10.1128/jb.175.11.3303-3316.1993 PMC204727

[64] genomics2ecology R package [Internet]. https://github.com/olgakamneva/genomics2ecology

[65] Kamneva OK. Data and scripts for the paper “Genome Composition of Microbes Predicts Their Co-Occurrence in the Environment” [Internet]. https://github.com/olgakamneva/Kamneva_2016

[66] Szklarczyk D, Franceschini A, Wyder S, Forslund K, Heller D, Huerta-Cepas J, et al STRING v10: protein-protein interaction networks, integrated over the tree of life. Nucleic Acids Res. 2015;43: D447–452. 10.1093/nar/gku1003 25352553 PMC4383874

[67] Dongen SM van. Graph clustering by flow simulation. 2001; http://dspace.library.uu.nl/handle/1874/848

[68] Pereira-Leal JB, Enright AJ, Ouzounis CA. Detection of functional modules from protein interaction networks. Proteins Struct Funct Bioinforma. 2004;54: 49–57. 10.1002/prot.10505 14705023

[69] van Dongen S, Abreu-Goodger C. Using MCL to extract clusters from networks. Methods Mol Biol. 2012;804: 281–295. 10.1007/978-1-61779-361-5_15 22144159

[70] McDonald D, Price MN, Goodrich J, Nawrocki EP, DeSantis TZ, Probst A, et al An improved Greengenes taxonomy with explicit ranks for ecological and evolutionary analyses of bacteria and archaea. ISME J. 2012;6: 610–618. 10.1038/ismej.2011.139 22134646 PMC3280142

[71] Greengenes [Internet]. ftp://greengenes.microbio.me/greengenes_release/gg_13_5/

[72] Cole JR, Wang Q, Fish JA, Chai B, McGarrell DM, Sun Y, et al Ribosomal Database Project: data and tools for high throughput rRNA analysis. Nucleic Acids Res. 2014;42: D633–642. 10.1093/nar/gkt1244 24288368 PMC3965039

[73] Markowitz VM, Chen I-MA, Palaniappan K, Chu K, Szeto E, Grechkin Y, et al IMG: the integrated microbial genomes database and comparative analysis system. Nucleic Acids Res. 2012;40: D115–D122. 10.1093/nar/gkr1044 22194640 PMC3245086

[74] Levandowsky M, Winter D. Distance between Sets. Nature. 1971;234: 34–35.

[75] Price MN, Dehal PS, Arkin AP. FastTree 2--approximately maximum-likelihood trees for large alignments. PloS One. 2010;5: e9490 10.1371/journal.pone.0009490 20224823 PMC2835736

[76] Legendre P, Legendre L. Numerical Ecology [Internet]. 1998 [cited 31 Dec 2015]. http://store.elsevier.com/Numerical-Ecology/P_-Legendre/isbn-9780080523170/

[77] Paradis E, Claude J, Strimmer K. APE: Analyses of Phylogenetics and Evolution in R language. Bioinformatics. 2004;20: 289–290. 14734327 10.1093/bioinformatics/btg412

[78] Oksanen J, Blanchet FG, Kindt R. Vegan: community ecology package. 2013. R Package Version. 2013; 2–0.

[79] Revell LJ. phytools: an R package for phylogenetic comparative biology (and other things). Methods Ecol Evol. 2012;3: 217–223.

