# Supplementary figures and images for: Genome composition and phylogeny of microbes predict their co-occurrence in the environment
Source: PLoS Comput Biol. 2017 Feb 2;13(2):e1005366. doi: 10.1371/journal.pcbi.1005366 (PMC5313232; doi:10.1371/journal.pcbi.1005366)

A

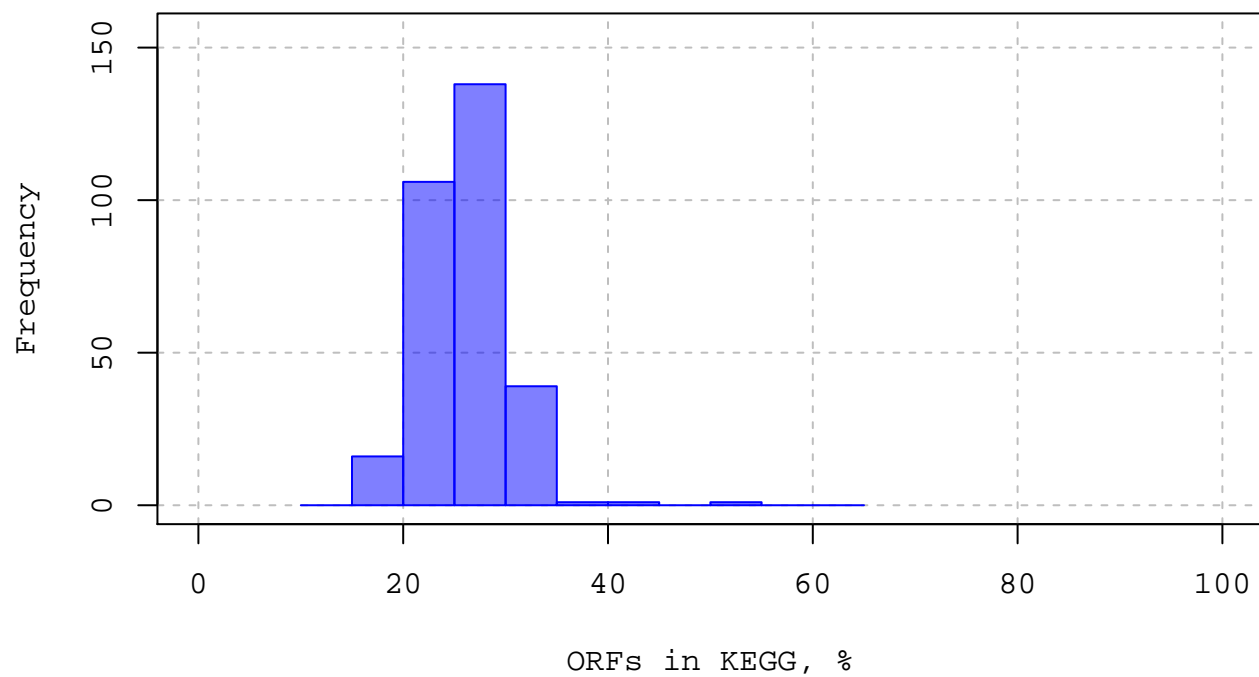

B

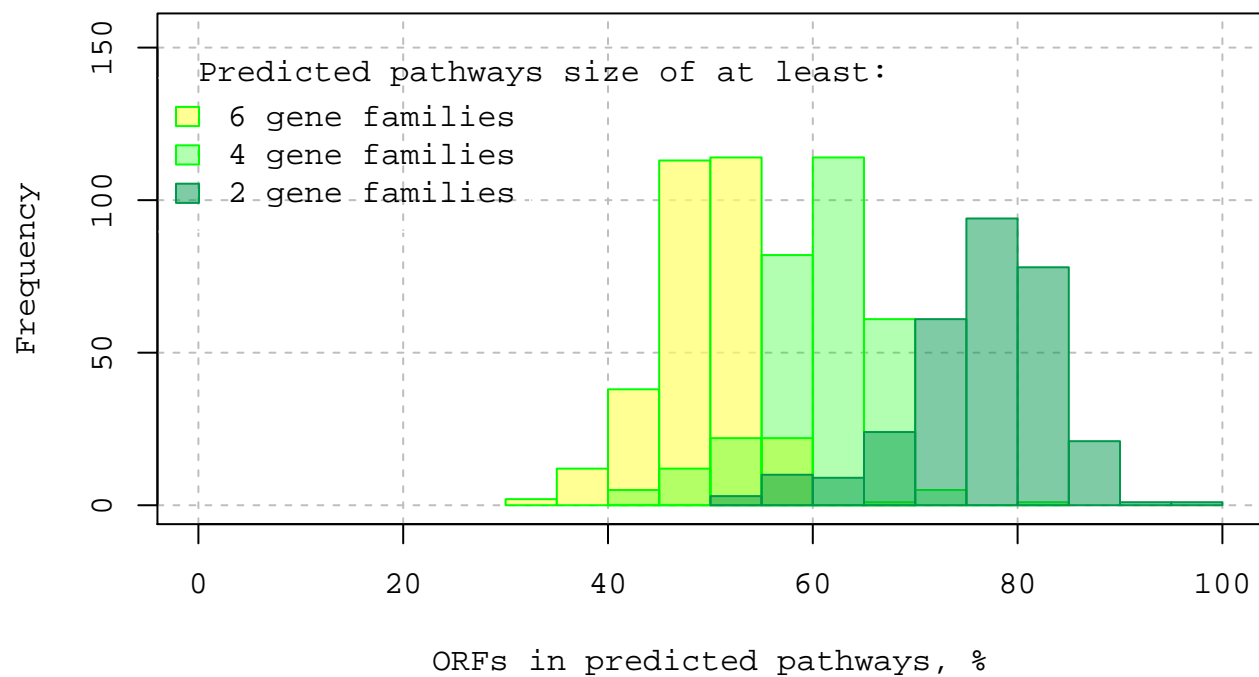

Supplement: S1 Fig — (A) Percent of the ORFs included in a KEGG pathways or (B) putative pathways predicted with MCL in 308 genomes from STRING from ecological dataset 1 which is introduced later in the paper. (PDF) [file pcbi.1005366.s001.pdf]

A

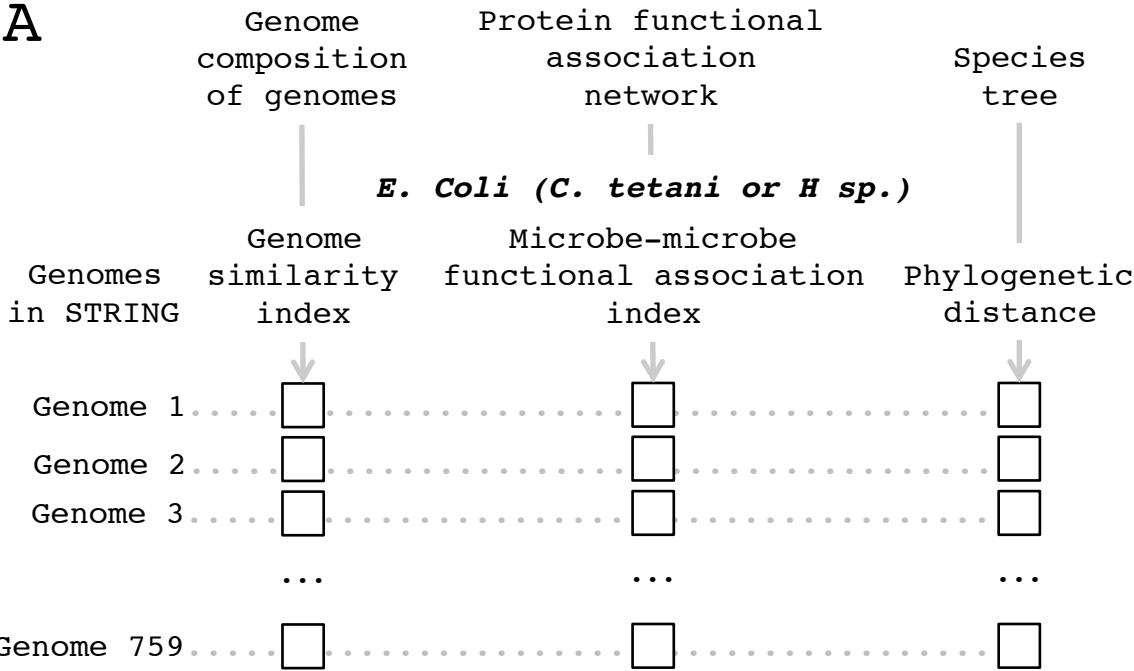

Scatterplots in Fig. 2

B

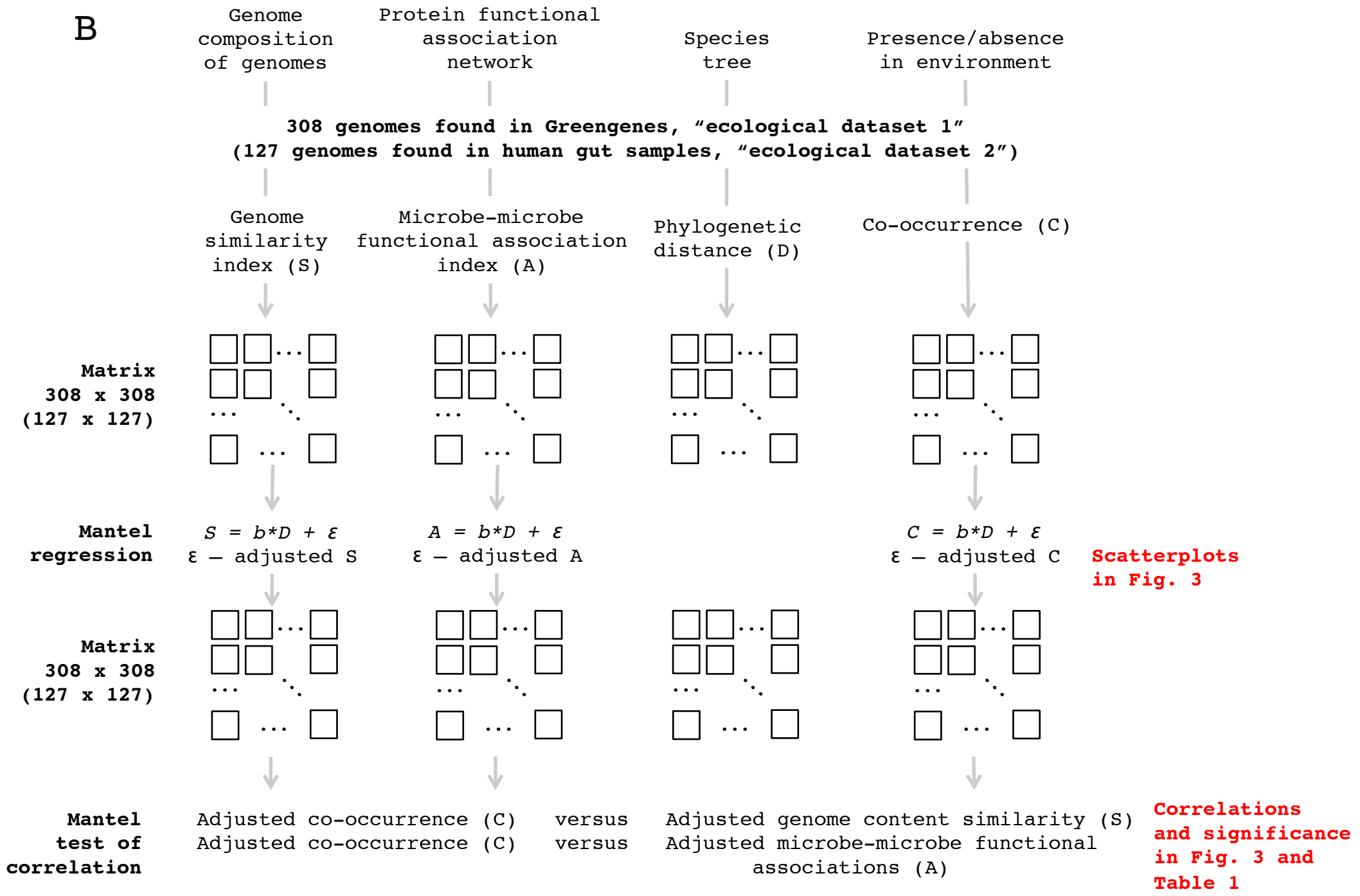

Supplement: S2 Fig — Graphical representation of statistical analysis workflow. (PDF) [file pcbi.1005366.s002.pdf]
